# Validation of microscopic observation drug susceptibility testing for rapid, direct rifampicin and isoniazid drug susceptibility testing in patients receiving tuberculosis treatment

**DOI:** 10.1111/1469-0691.12401

**Published:** 2013-11-21

**Authors:** J Coronel, M H Roper, C Herrera, C Bonilla, O Jave, C Gianella, I Sabogal, V Huancaré, E Leo, A Tyas, A Mendoza-Ticona, L Caviedes, D A J Moore, M Drancourt

**Affiliations:** 1) Laboratorio de Investigación de Enfermedades Infecciosas, Universidad Peruana Cayetano HerediaPeru; 2) Estrategia Sanitaria Nacional de Prevención y Control de la Tuberculosis, Ministerio de SaludPeru; 3) Universidad Nacional Mayor San MarcosPeru; 4) Laboratorio de Referencia Nacional de Micobacterias, Instituto Nacional de SaludLima, Peru; 5) LSHTM TB Centre and Faculty of Infectious and Tropical Diseases, London School of Hygiene and Tropical MedicineLondon, UK

**Keywords:** Drug susceptibility test, MODS, treatment failure, tuberculosis

## Abstract

Drug susceptibility testing (DST) is often needed in patients clinically failing tuberculosis (TB) therapy. Most studies of phenotypic direct drug susceptibility tests, such as microscopic observation drug susceptibility (MODS) tests, have been performed in patients not receiving TB treatment. The effect of ongoing TB treatment on the performance of MODS direct DST has not been previously explored, but patients failing such therapy constitute an important target group. The aim of this study was to determine the performance of MODS direct rifampicin and isoniazid DST in patients clinically failing first-line TB treatment, and to compare MODS direct DST with indirect proportion method DST. Sputa from 264 TB patients were cultured in parallel in Lowenstein–Jensen (LJ) and MODS assays; strains were tested for rifampicin and isoniazid susceptibility by the proportion method at the national reference laboratory. Ninety-three samples were culture-positive by LJ and MODS (concordance of 96%; kappa 0.92). With conventional MODS plate DST reading (performed on the same day as the sample is classified as culture-positive), the isoniazid DST concordance was 96.8% (kappa 0.89), and the concordance for rifampicin susceptibility testing was 92.6% (kappa 0.80). Reading of MODS DST plates 1 week after cultures had been determined to be culture-positive improved overall performance marginally—the isoniazid DST concordance was 95.7% (kappa 0.85); and the rifampicin DST concordance was 96.8% (kappa 0.91). Sensitivity for detection of multidrug-resistant TB was 95.8%. MODS testing provided reliable rifampicin and isoniazid DST results for samples obtained from patients receiving TB therapy. A modified DST reading schedule for such samples, with a final reading 1 week after a MODS culture turns positive, marginally improves the concordance with reference DST.

## Introduction

Direct drug susceptibility testing (DST) is a potentially powerful tool in the global battle against drug-resistant tuberculosis (TB). By circumventing the delay inherent in primary culture and strain isolation, and the additional workload and further delay associated with the establishment of secondary cultures for indirect DST, clinicians and their patients have access to this important information in a clinically useful time frame 1–4. Reservations about the validity of direct phenotypic DST for *Mycobacterium tuberculosis* are based upon a perceived need to control inoculum size. Overwhelming data obtained with several different methodologies have now demonstrated that, whereas this concern might be warranted for ethambutol and streptomycin (which tend to not perform very well in direct DST), direct DST shows high concordance with conventional indirect reference methods for both rifampicin and isoniazid 1,5–7.

The majority of published data on direct DST methodologies come from diagnostic studies on patients who are not receiving anti-TB therapy. For direct molecular resistance detection methods, there is no reason why TB treatment should influence test performance, and data can therefore be extrapolated to treated patients. However, for phenotypic direct DST, which depends on the growth of *M.  tuberculosis* in the absence and presence of carefully defined assay drug concentrations, it is biologically plausible that the presence of drug in the tissues and respiratory secretions of a TB patient receiving treatment might affect the performance of a direct drug susceptibility test.

Although the implementation of microscopic observation drug susceptibility (MODS) testing in Peru is aimed at providing pretreatment DST for all TB patients served by currently available MODS laboratories, there is an important demand for rapid DST in patients without baseline results who appear to be failing therapy. In order to validate the use of MODS testing for this indication, we undertook an evaluation in patients clinically failing to respond to first-line TB treatment. We compared the rifampicin and isoniazid direct DST results obtained with MODS testing with those obtained by the Peruvian national mycobacteria reference laboratory by using the reference standard, (indirect) proportion method testing, on agar.

## Materials and Methods

### Study setting, population, and recruitment

The study was performed in hospitals and health centres across Lima and Lima-Callao between September 2008 and December 2009. Patients failing to respond to first-line TB therapy (2RHZE_6_ 4HR_2_), defined as persistent symptoms (weight loss, cough, fever, and night sweats) and/or sputum smear non-conversion after 2 months of directly observed treatment, are routinely referred to respiratory physicians for evaluation, which usually includes conventional solid medium culture of a sputum sample followed by indirect DST by the proportion method on agar at the national TB reference laboratory. During the study period, physicians referred such patients to a member of the dedicated study team if a sputum sample was to be taken and the patient indicated willingness to participate in the study. Patients aged ≥18 years who agreed to participate after reading the study information sheet and discussing the study with the project nurse gave written informed consent, responded to a brief structured questionnaire, and provided a sample of sputum.

### Laboratory methods

Sputum samples were transported on the same day to the research laboratory at Universidad Peruana Cayetano Heredia (UPCH), where they were refrigerated until processing, usually within 24 h. Each sample was decontaminated by the conventional NaOH/*N*-acetyl-l-cysteine method, and auramine smear microscopy was performed on the concentrated pellet. The pellet was resuspended in supplemented Middlebrook 7H9 medium, and inoculated onto a Lowenstein–Jensen (LJ) slope and into the MODS assay as described previously elsewhere 1 (http://www.modsperu.org/MODS_user_guide.pdf).

MODS results were defined as either positive (at least two *M. tuberculosis* CFUs in each of the two drug-free control wells) or not positive, which included negative (no CFUs in either well) or indeterminate (*M. tuberculosis* CFUs observed in only one well or fewer than two in both wells) or contaminated cultures. For positive MODS cultures, the results of rifampicin and isoniazid DST were defined as susceptible (no growth seen), resistant (at least two CFUs seen) or indeterminate (only one CFU). The results of the reference proportion DST method, defined as susceptible or resistant, were available to clinicians for patient care.

### Data analysis

The primary outcome of interest was the concordance of direct MODS DST results with those from the indirect proportion DST method for rifampicin and isoniazid. We constructed 2 × 2 tables separately for rifampicin and isoniazid, and percentage concordance and kappa values were calculated (to determine percentage agreement beyond chance).

### Ethical review

The study protocol and informed consent form were reviewed and approved by the Instituto Nacional de Salud (National Institute of Health) and the National Tuberculosis Control Programme (ESNPCT) of the Peruvian Ministry of Health, and the ethics committee of UPCH, Lima, Peru.

## Results

### Culture

**Figure 1:**
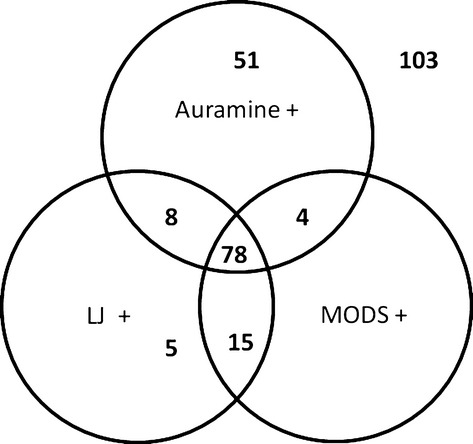
Smear and culture results for 264 samples from tuberculosis patients apparently failing first-line therapy. LJ, Lowenstein–Jensen; MODS, microscopic observation drug susceptibility.

A single sputum sample was collected and processed for each of 264 consecutive eligible patients failing first-line TB therapy; 123 (46.6%) were auramine smear-negative, of whom 20 (16.3%) were culture-positive by either or both LJ and MODS (Fig.1), the remainder (*n* = 103) being culture-negative by both MODS and LJ. Of the 141 smear-positive samples, 51 (36.2%) were culture-negative by both LJ and MODS. Of the smear-positive samples, 43.7%, 76.9% and 93.5% of acid-fast bacilli (AFB) 1+, AFB 2+ and AFB 3+ samples, respectively, were culture-positive by either or both methods (Table1).

**Table 1:** 2 × 2 tables of microscopic observation drug susceptibility (MODS) and Lowenstein–Jensen (LJ) culture positivity of 141 auramine smear-positive samples according to smear grading

		AFB 1+ *n* = 71				AFB 2+ *n* = 39				AFB 3+ *n* = 31	
		LJ				LJ				LJ	
		+	−			+	−			+	−
MODS	+	23	2	MODS	+	26	2	MODS	+	29	0
	−	6	40		−	2	9		−	0	2

AFB, acid-fast bacilli.

For AFB 1+ MODS: sensitivity, 79.3%; specificity, 95.2%; positive predictive value (PPV), 92.0%; negative predictive value (NPV), 87.0%.

For AFB 2+ MODS: sensitivity, 92.9%; specificity, 81.8%; PPV, 92.9%; NPV, 81.8%.

For AFB 3+ MODS: sensitivity, 100.0%; specificity, 100.0%; PPV, 100.0%; NPV, 100.0%.

### Concordance of MODS and LJ for TB detection in patients receiving TB treatment

In total, 93 samples were culture-positive by both MODS and LJ, 154 samples were not positive by either, and MODS and LJ results differed for 17 samples—concordance by this measure = 100 × (93 + 154)/(93 + 154 + 17) = 93.6% (kappa 0.86). However, among these 17 samples with discordant results, only ten were positive by one culture method and negative by the other; seven had positive or negative results by one method, and were either contaminated or indeterminate by the other (Table2). After the application of stricter criteria to limit the analysis to only those samples with two definitive (i.e. positive or negative) results, concordance was 96.0% (100 × (93 + 149)/(93 + 149 + 10); kappa 0.92).

**Table 2:** 3 × 3 table of tuberculosis detection by Lowenstein–Jensen (LJ) and microscopic observation drug susceptibility (MODS)

		LJ		
		Positive	Negative	Indeterminate
MODS	Positive	93	3	1^a^
	Negative	7	149	1^a^
	Indeterminate	6^b^	4^b^	

MODS: sensitivity, 93.0%; specificity, 98.0%; positive predictive value, 96.9%; negative predictive value, 95.5%.

a Culture contaminated.

b Mycobacterial growth seen, but lower than the threshold of two CFUs in two wells.

### Rifampicin and isoniazid DST

Proportion method results were available for 94 strains: 92 of the 93 that were culture-positive in LJ and MODS, plus two that were culture-positive only in MODS, from which the strain was sent for proportion method testing. By the proportion method, 14 of 94 strains (14.9%) were susceptible to both isoniazid and rifampicin, six of 94 (6.4%) were isoniazid-monoresistant, two of 94 (2.1%) were rifampicin-monoresistant, and 72 of 94 (76.6%) were resistant to both isoniazid and rifampicin.

### Conventional MODS DST well reading vs. the proportion method

For diagnostic samples from patients not receiving TB treatment, MODS DST wells are typically read and interpreted on the same day that the MODS drug-free well cultures become clearly positive. With this approach for samples with definitive results, the concordance for isoniazid susceptibility between MODS and the proportion method was 96.8% (kappa 0.89) (Table3), and the concordance for rifampicin susceptibility was 92.6% (kappa 0.80) (Table4).

**Table 3:** Isoniazid drug susceptibility testing by direct microscopic observation drug susceptibility (MODS) and indirect proportion method testing for patients failing treatment

Isoniazid		Proportion method	
		Susceptible	Resistant
MODS	Susceptible	15	2
	Resistant	1	76

For detection of isoniazid resistance with MODS testing: sensitivity, 97.4%; specificity, 93.8%; positive predictive value, 98.7%; negative predictive value, 88.2%.

**Table 4:** Rifampicin drug susceptibility testing by direct microscopic observation drug susceptibility (MODS) and indirect proportion method testing for patients failing treatment

Rifampicin		Proportion method	
		Susceptible	Resistant
MODS	Susceptible	20	7
	Resistant	0	67

For detection of rifampicin resistance with MODS testing: sensitivity, 90.5%; specificity, 100.0%; positive predictive value, 100%; negative predictive value, 74.1%.

### Delayed final MODS DST well reading vs. the proportion method

Although wells were read and DST results classified as described above, plates were retained and wells continued to be read for up to 3 weeks after cultures became positive. We analysed whether a modified (delayed) well interpretation improved concordance, allowing growth in drug-containing wells to be interpreted as resistant if it occurred within 7 or 14 days after TB growth was first identified in drug-free wells (and not only on the same day).

With this approach, the concordance for isoniazid susceptibility testing at 7 and 14 days after drug-free well cultures turned positive was 95.7% and 95.7%, respectively (kappa values of 0.85 and 0.85; Tables5 and 6), and the concordance for rifampicin susceptibility testing was 96.8% and 96.8%, respectively (kappa values of 0.91 and 0.91; Tables7 and 8).

**Table 5:** Isoniazid drug susceptibility testing by direct microscopic observation drug susceptibility (MODS) and indirect proportion method testing for patients failing treatment, with reading 1 week after culture positivity

Isoniazid		Proportion method	
		Susceptible	Resistant
MODS	Susceptible	14	2
	Resistant	2	76

For detection of isoniazid resistance with MODS testing: sensitivity, 97.4%; specificity, 87.5%; positive predictive value, 97.4%; negative predictive value, 87.5%.

**Table 6:** Isoniazid drug susceptibility testing by direct microscopic observation drug susceptibility (MODS) and indirect proportion method testing for patients failing treatment, with reading 2 weeks after culture positivity

Isoniazid		Proportion method	
		Susceptible	Resistant
MODS	Susceptible	14	2
	Resistant	2	76

For detection of isoniazid resistance with MODS: sensitivity, 97.4%; specificity, 87.5%; positive predictive value, 97.4%; negative predictive value, 87.5%.

**Table 7:** Rifampicin drug susceptibility testing by direct microscopic observation drug susceptibility (MODS) and indirect proportion method testing for patients failing treatment, with reading 1 week after culture positivity

Rifampicin		Proportion method	
		Susceptible	Resistant
MODS	Susceptible	20	3
	Resistant	0	71

For detection of rifampicin resistance with MODS: sensitivity, 95.9%; specificity, 100.0%; positive predictive value, 100.0%; negative predictive value, 87.0%.

**Table 8:** Rifampicin drug susceptibility testing by direct microscopic observation drug susceptibility (MODS) and indirect proportion method testing for patients failing treatment, with reading 2 weeks after culture positivity

Rifampicin		Proportion method	
		Susceptible	Resistant
MODS	Susceptible	19	2
	Resistant	1	72

For detection of rifampicin resistance with MODS: sensitivity, 97.3%; specificity, 95.0%; positive predictive value, 98.6%; negative predictive value, 90.5%.

The concordance for combined rifampicin/isoniazid DST results when MODS readings were taken at 0, 7 and 14 days after culture positivity is shown in Table9; the sensitivity for multidrug resistance (MDR) detection was 90.3%, 95.8% and 97.2%, respectively. Reading MODS DST results at day 7 after culture positivity increased the accuracy of detection of rifampicin resistance and MDR, at a minor cost in the specificity of isoniazid resistance detection (isoniazid resistance was slightly overestimated; see receiver operating characteristic curve data in Fig. S1).

**Table 9:** Combined rifampicin/isoniazid (RH) drug susceptibility testing (DST) and MODS DST results, with reading at 0 (conventional), 7 and 14 days after culture positivity; concordant results are highlighted in bold

		Proportion method result															
		INH S RIF S				INH R RIF S				INH R RIF R				INH S RIF R			
		*n* = 14				*n* = 6				*n* = 72				*n* = 2			
MODS result	INH	**S**	R	R	S	S	**R**	R	S	S	R	**R**	S	S	R	R	**S**
	RIF	**S**	S	R	R	S	**S**	R	R	S	S	**R**	R	S	S	R	**R**
Day	0	**13**	1			1	**5**			1	6	**65**					**2**
	7	**13**	1			1	**5**			1	2	**69**				1	**1**
	14	**13**	1			1	**4**	1		1	1	**70**				1	**1**

INH, isoniazid; R, resistant; RIF, rifampicin; S, susceptible.

## Discussion

The key finding of this study was that the results of MODS isoniazid and rifampicin direct DST are as reliable for samples from patients receiving TB treatment as for samples obtained before patients begin therapy. Importantly, the sensitivities and specificities of MODS results for detection of rifampicin monoresistance, isoniazid monoresistance and MDR were comparable to those reported in previous studies of MODS test performance with samples from untreated patients 6, supporting the use of MODS testing not only in those suspected of having TB and in newly diagnosed TB patients prior to treatment initiation, but also in patients failing treatment. Furthermore, clinicians and laboratory staff can now be assured of the validity of MODS DST when used for patients who have recently commenced therapy without baseline DST, provided that they are still culture-positive.

In MODS cultures of samples from untreated patients, breakthrough growth is occasionally seen 1 or 2 weeks after cultures are initially positive, but this does not correspond with drug resistance; however, in this study of patients receiving TB treatment, we observed that some late breakthrough growth occurred in strains initially believed to be monoresistant, so late growth was monitored. The sensitivity of resistance detection was marginally improved when MODS DST wells were re-read 1 week after the initial culture was deemed to be positive; this contrasts with findings when MODS testing is performed on samples from untreated patients, when accuracy diminishes with delayed reading. This might be because of a relative blunting of growth of mycobacteria, owing to the presence of anti-TB drugs in the sputum sample or exposure to TB drugs *in vivo*. Deferring the final MODS DST reading of drug-containing wells for an additional week for samples from treated patients only may be indicated when the source patient's clinical history is known; however, the utility of the modification depends on the availability of accurate clinical information. Moreover, the marginal benefit gained may not justify the additional complexity of having different reading schedules for different sample types. Laboratories might prefer to explore the incremental benefit of the delayed final reading during the MODS accreditation process 8.

An important strength of this study was the use of patients from a crucial target group for DST, namely those TB patients apparently failing first-line therapy who had not undergone baseline DST. MODS testing can accurately and quickly (usually within 7–14 days) indicate whether more extended DST and an interim switch to a standardized MDR treatment regimen is needed. Data on the use of other non-commercial phenotypic tests for direct DST in treated TB patients should also be gathered, as these methodologies hold considerable promise for widening the availability of affordable rifampicin and isoniazid DST, a gateway to appropriate treatment for the more than half a million patients with multidrug-resistant TB around the globe 9.

## Supporting Information

Figure S1a Receiver operating characteristic (ROC) curve indicating the effects of delayed (7 and 14 day) MODS DST reading upon the sensitivity and specificity of detection of isoniazid, rifampicin and combined isoniazid and rifampicin resistance (MDR) in patients failing TB treatment.

Figure S1b Detail of ROC curve from Fig. S1a.
