# Supplementary figures and images for: Validation of microscopic observation drug susceptibility testing for rapid, direct rifampicin and isoniazid drug susceptibility testing in patients receiving tuberculosis treatment
Source: Clin Microbiol Infect. 2013 Nov 21;20(6):536–41. doi: 10.1111/1469-0691.12401 (PMC4302318; doi:10.1111/1469-0691.12401)

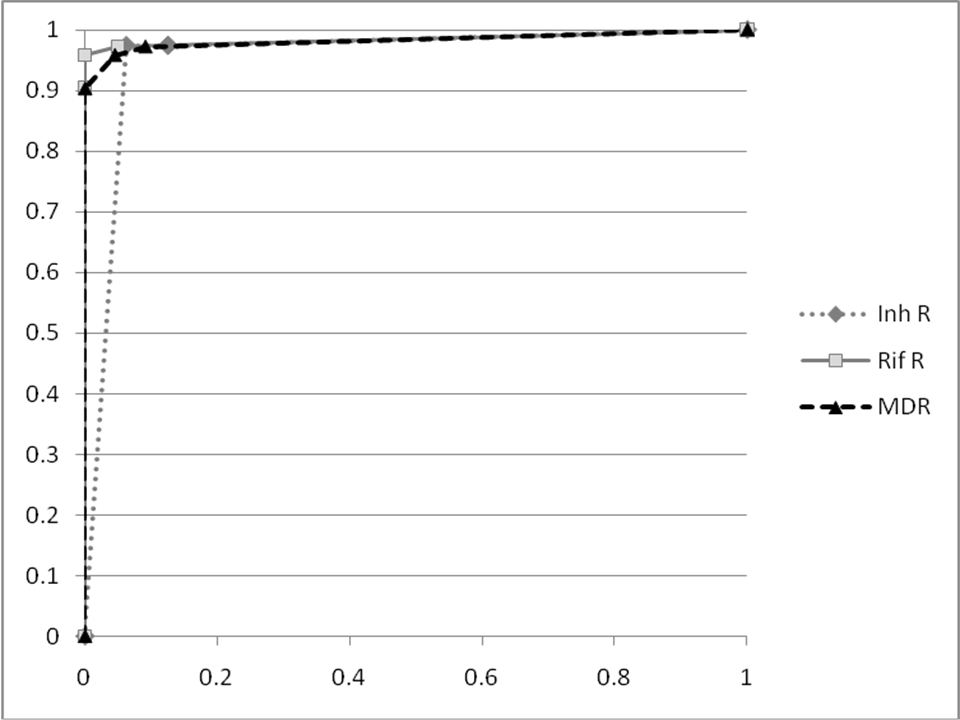

Supplement: Figure S1a — Receiver operating characteristic (ROC) curve indicating the effects of delayed (7 and 14 day) MODS DST reading upon the sensitivity and specificity of detection of isoniazid, rifampicin and combined isoniazid and rifampicin resistance (MDR) in patients failing TB treatment. [file clm0020-0536-sd1.tif]

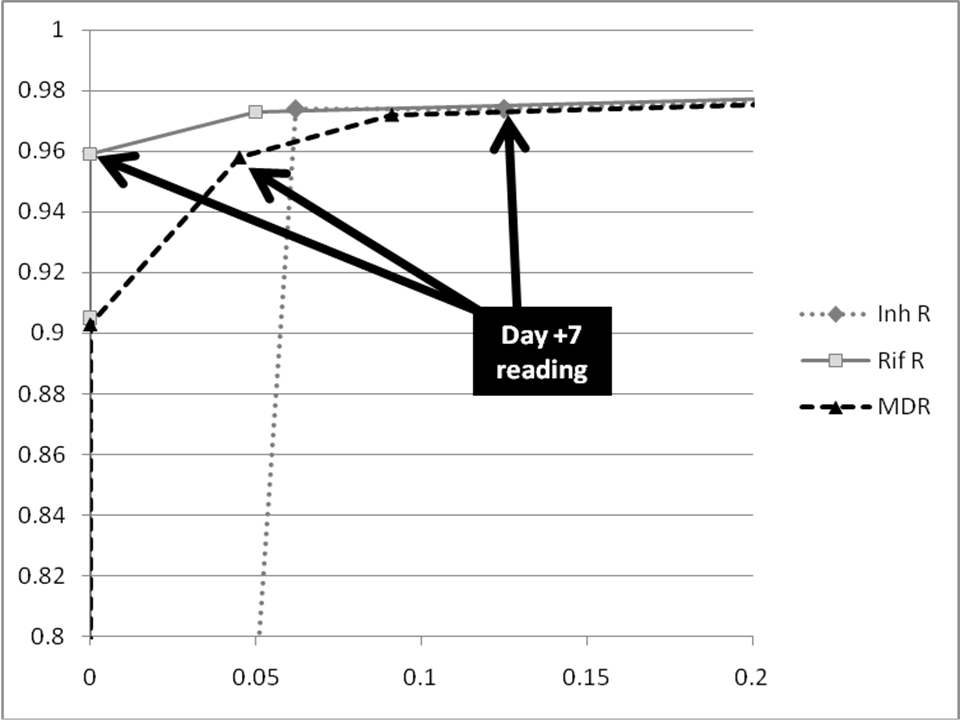

Supplement: Figure S1b — Detail of ROC curve from Fig. S1a. [file clm0020-0536-sd2.tif]
